# Supported Gold Nanoparticles as Catalysts in Peroxidative and Aerobic Oxidation of 1-Phenylethanol under Mild Conditions

**DOI:** 10.3390/nano10010151

**Published:** 2020-01-15

**Authors:** Ekaterina Pakrieva, Ana P. C. Ribeiro, Ekaterina Kolobova, Luísa M. D. R. S. Martins, Sónia A. C. Carabineiro, Dmitrii German, Daria Pichugina, Ce Jiang, Armando J. L. Pombeiro, Nina Bogdanchikova, Vicente Cortés Corberán, Alexey Pestryakov

**Affiliations:** 1 Research School of Chemistry & Applied Biomedical Sciences, National Research Tomsk Polytechnic University, Lenin Av. 30, 634050 Tomsk, Russia; ekaterina_kolobova@mail.ru (E.K.); germandmitry93@gmail.com (D.G.); pestryakov2005@yandex.ru (A.P.); 2 Centro de Química Estrutural, Instituto Superior Técnico, Universidade de Lisboa, Av. Rovisco Pais, 1049-001 Lisboa, Portugal; apribeiro@tecnico.ulisboa.pt (A.P.C.R.); luisammartins@tecnico.ulisboa.pt (L.M.D.R.S.M.); pombeiro@tecnico.ulisboa.pt (A.J.L.P.); 3 Instituto de Catálisis y Petroleoquímica, Consejo Superior de Investigaciones Científicas, Marie Curie 2, 28049 Madrid, Spain; vcortes@icp.csic.es; 4 Department of Chemistry, Moscow State University, 1–3 Leninskiye Gory, 119991 Moscow, Russia; dashapi@mail.ru (D.P.); jiangce804@gmail.com (C.J.); 5 Centro de Nanocienciasy Nanotecnología, Universidad Nacional Autónoma de México, Ensenada 22800, Mexico; nina@cnyn.unam.mx

**Keywords:** gold, heterogeneous catalysis, alcohol oxidation, 1-phenylethanol, TBHP, DFT

## Abstract

The efficiency of Au/TiO_2_ based catalysts in 1-phenylethanol oxidation was investigated. The role of support modifiers (La_2_O_3_ or CeO_2_), influence of gold loading (0.5% or 4%) and redox pretreatment atmosphere, catalyst recyclability, effect of oxidant: *tert*-butyl hydroperoxide (TBHP) or O_2_, as well as the optimization of experimental parameters of the reaction conditions in the oxidation of this alcohol were studied and compared with previous studies on 1-octanol oxidation. Samples were characterized by temperature-programmed oxygen desorption (O_2_-TPD) method. X-ray photoelectron spectroscopy (XPS) measurements were carried out for used catalysts to find out the reason for deactivation in 1-phenylethanol oxidation. The best catalytic characteristics were shown by catalysts modified with La_2_O_3_, regardless of the alcohol and the type of oxidant. When O_2_ was used, the catalysts with 0.5% Au, after oxidative pretreatment, showed the highest activity in both reactions. The most active catalysts in 1-phenylethanol oxidation with TBHP were those with 4% Au and the H_2_ treatment, while under the same reaction conditions, 0.5% Au and O_2_ treatment were beneficial in 1-octanol oxidation. Despite the different chemical nature of the substrates, it seems likely that Au^+^(Au^δ+^) act as the active sites in both oxidative reactions. Density functional theory (DFT) simulations confirmed that the gold cationic sites play an essential role in 1-phenylethanol adsorption.

## 1. Introduction

Alcohol oxidation is one of the most important transformations in industrial organic chemistry and a challenging process in terms of green chemistry [1,2]. Traditional methods involve the use of toxic, expensive, stoichiometric metal oxidants and harmful organic solvents, and often require harsh reaction conditions [3,4]. Catalysis research is working towards favourable solutions to these problems through the development of effective heterogeneous catalysts for environment friendly applications.

One example is the development and study of catalytic systems for the oxidation of 1-phenylethanol, which is an aromatic alcohol with high reactivity. The main oxidation product of its oxidation is acetophenone (methylphenylketone, C_6_H_5_COCH_3_). Acetophenone is used in perfumes, soaps and creams, as well as a flavouring substance in food, soft drinks and tobacco. It is also used as a solvent and has a sleeping effect, important in the manufacture of medicines.

There are many studies reported for 1-phenylethanol oxidation, using both homogeneous and heterogeneous catalysis (Table 1). From the summarized results, it can be concluded that the catalytic oxidation of this alcohol, a representative of cyclic alcohols, can be carried out under mild conditions: using green oxidizing agents such as oxygen, air and peroxides, both with a solvent and in its absence, in moderate temperature ranges: from room temperature to 150 °C and, in most cases, at atmospheric pressure. Also, when using peroxides, a new trend is the reaction under the influence of microwave heating, which, in comparison with the traditional process, allows obtaining high yields in a short time. 

Some disadvantages exist for homogeneous catalysts, namely the inability to reuse them, and the need for addition of bases and radicals, which researchers often apply to increase the yield of acetophenone. However, an exception is the work where some of us were able to synthesize catalysts based on iron complexes with low solubility, which were efficiently reused [9].

When heterogeneous base metals catalysts were used, selective oxidation of 1-phenylethanol at room temperature was possible, however, a large catalyst load and a long reaction time up to 24 h were required [13]. Catalysts based on palladium, silver, and ruthenium, exceeded the activity of previous catalyst systems, as expected, and were not deactivated during the recycling tests [16,17]. However, the disadvantages in the case of silver and palladium catalysts would include the formation of by-products [16,18,19], and a high ratio of ruthenium to alcohol needed when using ruthenium catalysts [20].

Gold-containing systems have been extensively investigated in this process [19,21,22,23,24,25,26,27,28,29,30,31,32]. The main feature of these systems is their high activity and selectivity; however, there is a tendency to their gradual deactivation due to an increase in the size of gold nanoparticles (Au NPs) during reaction and recycling tests. Also, in most cases, the authors suggest that highly dispersed gold in the metallic state is responsible for the excellent activity in the oxidation of 1-phenylethanol [24,26,27,29,31]. However, there are also supporters of the cationic nature of gold active centres in this process [23,28,33] and Liang et al. [25] considered negative charged gold as an active site.

Unfortunately, there are very few works in which the mechanism of both aerobic and especially peroxidative oxidation of 1-phenylethanol is proposed. Furthermore, when using TBHP as the oxidizing agent, the role of the catalyst is attributed to the decomposition of this oxidant into radicals responsible for the direct oxidation of alcohol [34,35]. In general, it can be concluded that supported gold-containing systems are effective catalysts in the oxidation of 1-phenylethanol; however, even in the oxidation of such a reactive alcohol, the study of the mechanism of formation of the active surface of gold-containing systems responsible for excellent catalytic performance is still the subject of numerous discussions.

Previously, in a series of our works [36,37,38,39], it was shown that 1-octanol, a representative of the less reactive alcohols, whose physical properties impose constraints to implementation of green chemistry approaches, could be effectively oxidized under mild conditions and using Au/(Me*_x_*O*_y_*)/TiO_2_ catalysts. It was found that the formation of an active surface responsible for the catalytic properties is strictly dependent on the modifying additives used for improving metal-support interaction, a way to transform and stabilize positively charged gold active sites. It would be expected that such catalysts would exhibit higher catalytic activity in the oxidation of more reactive alcohols, such a 1-phenylethanol, which lead us to carry out the present study. Moreover, this work should also allow comparing the catalytic behaviours towards different types of primary alcohols, one inactivated (alkyl) and another activated (bearing an aromatic ring), represented by 1-octanol and 1-phenylethanol, respectively.

Therefore, in this work, we continued to investigate Au/TiO_2_ based catalysts, unmodified and modified with lanthana and ceria, aiming to assess their performance in 1-phenylethanol oxidation under mild conditions. We also wanted to compare the results with those previously obtained for 1-octanol oxidation, using the same catalysts, to find out whether the nature of the active sites of catalysts in 1-phenylethanol oxidation is the same as in 1-octanol, depending on a number of factors: the influence of the nature of the oxidizing agent, support nature, the pretreatment atmosphere and gold content. Additionally, to find a theoretical conclusion about the effect of gold cationic sites on alcohol activation, DFT simulations were performed.

## 2. Materials and Methods

Titania P25 (nonporous, 70% anatase and 30% rutile, particle size: 21 nm, purity: 99.5%, Evonik Degussa GmbH, (Essen, Germany) was used as the starting support and was modified by impregnation with aqueous solutions of La(NO_3_)_3_·6H_2_O or Ce(NO_3_)_3_·6H_2_O (Merck, Darmstadt, Germany) in a molar ratio Ti/Me (La, Ce) = 40. Au was loaded on the supports with a nominal loading of 0.5 and 4 wt.% using HAuCl_4_·3H_2_O (Merck, Darmstadt, Germany) as precursor, by deposition-precipitation with urea (Merck, Darmstadt, Germany) in the absence of light following the previously reported procedure [40,41]. Briefly, the gold precursor (4.2 × 10^−3^ M) and urea (0.42 M) were dissolved in distilled water, and thereafter the support was added to the solution. The resulting mixture was heated to 80 °C and kept at constant temperature for 16 h, with stirring. The initial pH was 2.4. The pH was adjusted to 7.5 by the end of gold deposition. After the deposition–precipitation procedure, the samples were centrifuged (11,000 rpm, 15 min), washed with water then centrifuged four times, and dried under vacuum 2 h at 80 °C. After drying, the samples were stored at room temperature in a desiccator under vacuum, away from light, in order to prevent any alteration.

Catalysts were previously characterized by adsorption of N_2_ at −196 °C on a Micromeritics Tristar 3000 Apparatus, Micromeritics Instrument Corporation (Norcross, GA, USA), X-ray diffraction (XRD) on a Philips XPert PRO diffractometer (Amsterdam, Netherlands), X-ray photoelectron spectroscopy (XPS), on a ESCALAB 200A, VG Scientific (Waltham, MA, USA), energy-dispersive X-ray spectroscopy (EDX), transmission electron microscopy (TEM), as well as scanning transmission electron microscopy-high angle annular dark field (STEM-HAADF) using one single microscope (JEOL JEM-2100F, JEOL Ltd., Tokyo, Japan), temperature programmed reduction (H_2_-TPR) on a ChemiSorb 2750, (Micromeritics Instrument Corporation, Norcross, GA, USA) [38,39,42], and diffuse reflectance Fourier transform infrared (DRIFT) spectra of adsorbed CO [43] on a Bruker EQUINOX 55/S FTIR spectrometer (Billerica, MA, USA).

In this work, temperature-programmed oxygen desorption (O_2_-TPD) was used to assess the nature of the interaction of oxygen with the surface of the catalyst and the support. All experiments were performed on a “Chemosorb” chemisorption analyzer (Neosib, Novosibirsk, Russia) equipped with a thermal conductivity detector (TCD), which was calibrated with O_2_ prior analysis. A sample (0.2 g) pretreated at 300 °C in helium flow (60 mL/min) was saturated with oxygen at 40 °C for 1 h. Oxygen desorption was carried out in a helium stream from 40 to 650 °C with a heating rate of 20 °C/min.

The used (i.e., after the reaction test) catalysts were also characterized by X-ray photoelectron spectroscopy. Therefore, the surface composition and the chemical state of gold were determined by XPS analysis, performed on an ESCALAB 200A spectrometer (VG Scientific, Waltham, MA, USA) using Al Kα radiation (1486.6 eV). A pass-energy of 40 eV and a step size of 0.1 eV/step were selected. The charge effect was corrected using the C1s peak as a reference (binding energy of 285 eV). The CASA XPS software (version 2.3.15, CASA Software Ltd, Teignmouth, UK, http://www.casaxps.com/) was used for data analysis.

The catalytic properties for 1-phenylethanol oxidation were studied using catalyst samples either without treatment or after pre-treatment at 300 °C in H_2_ or O_2_ atmosphere. Therefore, catalysts samples will be denoted hereinafter as *n%* Au/(Me*_x_*O*_y_*)/TiO_2__X, where *n* is the gold content in wt.%, Me is the modifier metal (La or Ce) and X indicates the applied pretreatment atmosphere (O_2_ or H_2_). The catalytic tests were carried out in a batch reactor operated under atmospheric conditions at the temperature 80 °C for 6 h. The stirring reaction mixture was as follows: 1.27 µmol of Au were introduced in 1-phenylethanol (6 mmol) as the substrate, TBHP (70% *v/v* aqueous solution, 14.6 mmol) as the oxidizing agent, in a base- and solvent-free medium. 

After the reaction test, the mixture was allowed to cool down to room temperature. Typically, to conduct the product analysis, 10 µL of benzaldehyde (internal standard) and 1 mL of MeCN were added to 100 µL of such reaction mixture. The resulting sample was centrifuged for 15 min and analyzed by gas chromatography (GC) using the internal standard method. Blank tests indicated that only traces (4%) of ketone were generated. Chromatographic analyses were undertaken using a GC 8000 series gas chromatograph (Fisons Instruments, Loughborough, UK) equipped with a BP-20 (WAX) capillary column (SGE Analytical Science Europe Ltd, Milton Keynes, UK) and a flame ionization detector (FID) detector (Fisons Instruments, Loughborough, UK). Molar yield (%) was defined based on substrate, i.e., moles of product per 100 mol of substrate, determined by GC. Attribution of peaks was made by comparison with chromatograms of genuine samples and, in some cases, by gas chromatography mass-spectrometry (GC–MS) analyses with He as the carrier gas using a Clarus 600C instrument (Perkin Elmer, Waltham, MA, USA), equipped with a 30 m × 0.22 mm × 25 µm BPX5 (SGE Analytical Science Europe Ltd, Milton Keynes, UK) capillary column. According to the GC-MS acetophenone was the only product in both aerobic and peroxidative oxidation of 1-phenylethanol.

To adequately compare the catalytic results of aerobic oxidation of 1-phenylethanol with those of 1-octanol, the conditions selected for the oxidation of 1-phenylethanol with molecular oxygen were as follows: alcohol/gold ratio (*R*) = 100 mol/mol, 25 mL of a 0.1 M solution of 1-phenylethanol in mesitylene, O_2_ flow = 30 mL/min, stirring 800 rpm. And to compare the catalytic results of the peroxidative oxidation of 1-octanol with of those of 1-phenylethanol, the conditions selected for the peroxidative oxidation of 1-octanol were as follows: *R* = 5000 mol/mol, *T* = 80 °C, TBHP/alcohol = 2.43 mmol/mmol, stirring 800 rpm, without solvent or base added. In the absence of support/catalyst in the reaction mixture no activity was observed in both aerobic and peroxidative oxidation of 1-octanol.

To perform the recycling experiments, the used sample was separated from the reaction mixture by centrifugation (5000 rpm, 15 min) and decantation, washed 4 times with 5 mL of acetonitrile and dried at 50 °C to constant weight. It was then reused for the oxidation test as described above.

The adsorption of 1-phenylethanol on gold nanoparticles was simulated in a scalar-relativistic approach using the density functional (DFT) method PBE [44]. The tetrahedral Au_20_ and Au_20_^+^ clusters are considered as models of gold nanoparticles. The Au_20_ cluster has been obtained experimentally [45] and is a popular model for studying structural effects in catalysis [46,47]. Because the cluster has atoms located at the top, on the facet and on the edge and having different coordination number, we can effectively apply this model to study the structural effects in the adsorption of phenylethanol. A cationic gold cluster was obtained by removing one electron from Au_20_ with subsequent optimization of the structure. The effect of the support was ignored as a first approximation.

The interaction of 1-phenylethanol or 2-phenylethanol with Au_20_ cluster was simulated:

Au_20_ + CH_3_–CH(OH)–Ph → (CH_3_–CH(OH)–Ph)Au_20_ (1)

Au_20_ + OH–CH_2_–CH_2_–Ph → (OH–CH_2_–CH_2_–Ph)Au_20_ (2)

Different coordination modes of alcohol on Au_20_ by the OH group or the phenyl fragment were considered. The structures of (OH–CH_2_–CH_2_–Ph)Au_20_ isomers were optimized, and the total energies of the reagents and products were calculated taking into account the energy of zero vibrations. The change in total energy and standard enthalpies of the reaction (1) were determined according to the formula:

Δ*E* = *E*(phenylethanol–Au_20_) − *E*(Au_20_) − *E*(phenylethanol) (3)

The calculations were performed in the PRIRODA program (version 17, Russia) [48] using a Lomonosov supercomputer [49].

## 3. Results and Discussion

### 3.1. Catalytic Results

#### 3.1.1. Peroxidative Oxidation of 1-Phenylethanol

**Scheme 1:**
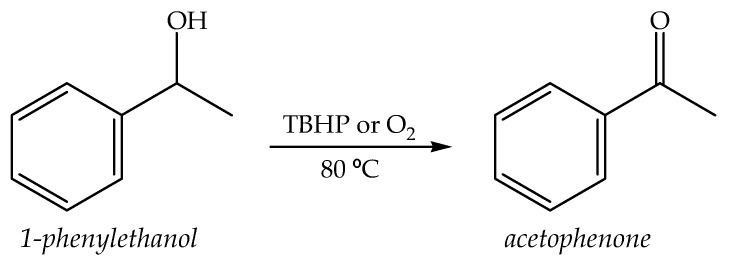
Oxidation of 1-phenylethanol over Au/(Me*_x_*O*_y_*)/TiO_2_ catalysts.

Since we had the goal of finding out the same active sites in both processes, the reaction conditions should be the same. Therefore, as in the case of 1-octanol oxidation, all experiments on both peroxidative and aerobic oxidation of 1-phenylethanol were carried out at a temperature of 80 °C (conventional heating) (Scheme 1). 

We started the catalytic experiments with TBHP as oxidizing agent with a very low catalyst loading equal to the ratio of 1-phenylethanol/Au of 5000 and in no-solvent conditions. The results are presented in Table 2. As expected, both unmodified and modified catalysts proved to be much more active in the oxidation of 1-phenylethanol than in that of 1-octanol. Taking into account the high alcohol/gold ratio, the catalysts, even in the as-prepared state, reached more than 50% of acetophenone yield after 6 h of reaction, despite the low activity in the first hours (Scheme 1). As in the oxidation of 1-octanol [38,39], the sample modified with lanthanum oxide demonstrated the best catalytic performance (Table 2): 98% yield of acetophenone was achieved already in 1 h when 4% Au/La_2_O_3_/TiO_2_ pretreated in H_2_ was used. It should also be noted that the activity of the studied catalysts increased with increasing gold content.

However, it should be emphasized that the supports also showed some activity under these conditions, as the yield of acetophenone increased with time and depended on the support nature (Entries 2–4, Table 2). In the absence of a catalyst or support, the formation of a small amount of acetophenone was also observed (Entry 1, Table 2). For the oxidation of 1-phenylethanol with molecular oxygen, no activity was observed when using supports only, and neither in the absence of a support/catalyst, as will be seen below. This shows the very good activity of TBHP, which, when decomposed by heating or by reaction with a metal (see Introduction), forms radicals (t-BuO·, t-BuOO·), responsible for the direct oxidation of alcohols [34]. Therefore, in the referred work, 97% acetophenone was obtained using 6 equivalents of TBHP at 100 °C in 24 h, without adding any catalysts or bases [35].

When using the La-modified sample, which showed a medium activity in 1-phenylethanol peroxidative oxidation, direct dependence of the yield of acetophenone on catalyst loading was observed (Table 3). Thus, 100% acetophenone was reached already after 15 min of reaction when the total gold amount was increased from 1.27 to 10 µmol. It should be noted that experiments using hydrogen peroxide (30% aqueous solution) as oxidant were not effective.

In the case of the 4% Au/La_2_O_3_/TiO_2__H_2_ catalyst, only 11% of the product yield was achieved after 6 h of reaction, under the same conditions used with TBHP (*T* = 80 °C, 6 mmol 1-phenylethanol, 14.6 mmol H_2_O_2_, 1.27 µmol of Au). With the same catalyst, a 98% yield of acetophenone was obtained after 1 h using TBHP (Entry 20, Table 2). Thus, conditions close to the principles of green chemistry were selected to compare with the above-mentioned oxidation process of 1-octanol: a solvent-free oxidation of 1-phenylethanol with Au/(Me*_x_*O*_y_*)/TiO_2_ catalysts, for 6 h, at *T* = 80 °C, using a minimum gold loading (1.27 µmol), and TBHP as a green oxidant, without any additives.

#### 3.1.2. Aerobic Oxidation of 1-Phenylethanol

After replacing TBHP with molecular oxygen, keeping the same alcohol/gold ratio (*R* = 5000), we did not observe any conversion, even after 6 h of reaction (Table 4). 

The next step was to investigate the effect of the alcohol/gold ratio. With a ten-fold increase in catalyst loading (*R* = 500), 50% conversion was observed after 6 h of reaction. A complete conversion of 1-phenylethanol, using this catalyst, could be achieved only at *R* = 100, with 98% acetophenone yield being reached after just 30 min of reaction.

**Figure 1:**
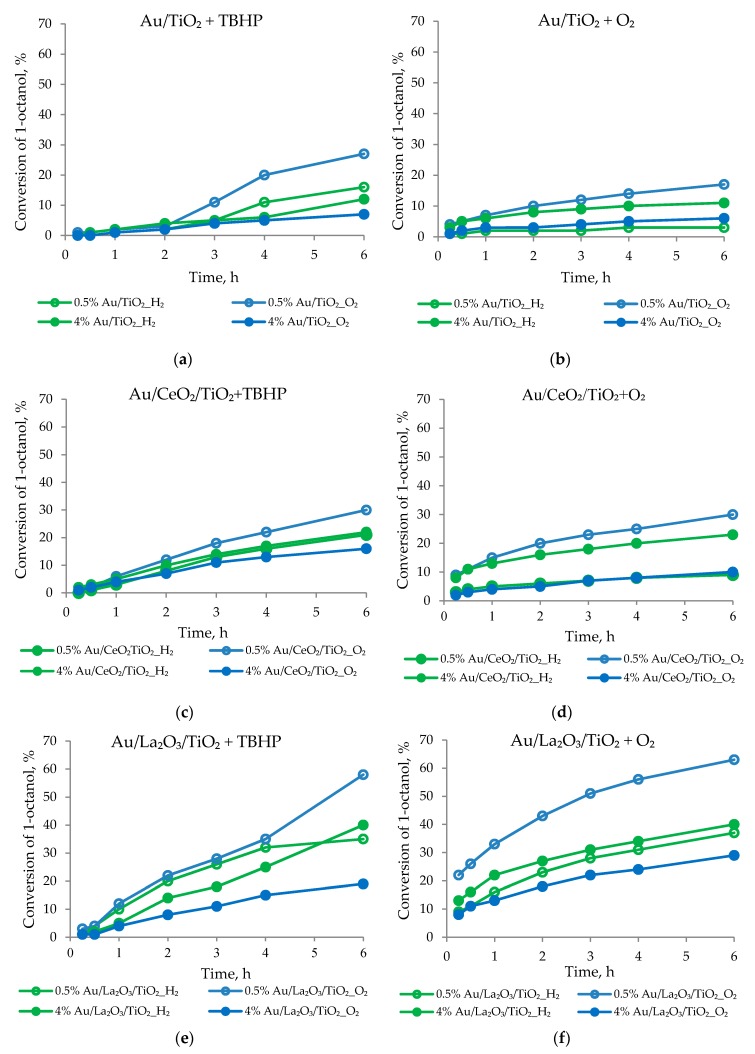
Time evolution of catalytic peroxidative (**a**,**c**,**e**) and aerobic (**b**,**d**,**f**) oxidation of 1-octanol. Reaction conditions of solvent-free peroxidative oxidation: *R* = 5000, TBHP: 1-octanol = 2.43; *T* = 80 °C, stirring = 800 rpm. Reaction conditions of *aerobic* oxidation: *R* = 100, 0.1 M 1-octanol in n-heptane; *T* = 80 °C, 30 mL/min O_2_, stirring = 800 rpm.

Such a different behaviour in the catalytic activity probably lies in the different oxidative capacity of oxygen and TBHP. Thus, the catalytic activity in the aerobic oxidation of 1-phenylethanol of the remaining catalysts was studied with R = 100. As it can be seen in Table 5, catalysts with a low gold content with an oxidative pretreatment were the most active, as in the case of 1-octanol (Figure 1b,e,f) [39].

#### 3.1.3. Peroxidative and Aerobic Oxidation of 1-Octanol

**Scheme 2:**
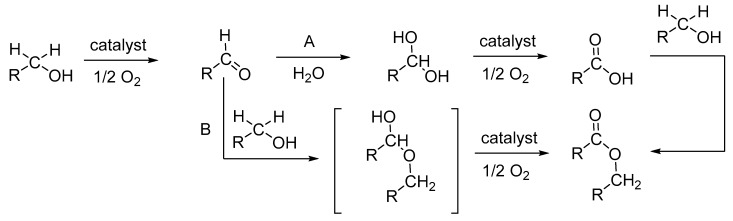
Possible reaction pathways for the oxidation of 1-octanol on supported gold catalysts (Adapted from [36,39]).

Since this work also aimed at making a comparison with previous results obtained for the oxidation of 1-octanol, we also studied the effects of the oxidizing agent in the oxidation of this substrate. The trends of catalytic behaviour of the catalysts in the peroxidative and aerobic oxidation of 1-octanol were similar in terms of activity (Figure 1). The most active catalysts were those with low Au loading, after an oxidative pretreatment. Regarding selectivity, it is important to note that when using molecular oxygen as an oxidizing agent, a different behaviour is observed in the distribution of oxidation products, depending on the pretreatment, the nature of the support and the gold content [39]. However, when TBHP was used as oxidizing agent, in the absence of a solvent, the main product, in all cases, was acid with a small amount of ester (up to 20%). This is probably due to the peroxide used as oxidizing agent which decomposed producing water [34], that is needed for octanoic acid formation, according to the Scheme 2 (route A) from [36,39].

Therefore, according to all catalytic results, under comparable reaction conditions, 1-phenylethanol can be much more efficiently and selectively oxidized over Au/Me*_x_*O*_y_*/TiO_2_ catalysts than 1-octanol. Moreover, the best results in the oxidation of both alcohols were achieved using TBHP.

In all cases, the best catalytic characteristics were shown by catalysts modified with lanthanum oxide, regardless of the alcohol and the type of oxidizing agent. Also, when a solvent was used and molecular oxygen was present as an oxidizing agent, the catalysts with the lowest gold content after oxidative pretreatment showed the highest activity in both 1-phenylethanol and 1-octanoloxidation. 

The only difference was that under no-solvent peroxidative conditions, the most active catalysts in 1-phenylethanol oxidation are those with a high load of gold and the hydrogen treatment, while under the same reaction conditions, low gold loading and oxygen treatment were beneficial in 1-octanol oxidation.

### 3.2. Catalyst Characterization

Catalysts were previously characterized by several techniques [38,39,42,43]. The N_2_ adsorption and EDX analysis (Table 6) showed that the textural properties and the content of gold cannot be the reason for the different catalytic behaviour observed for the catalysts. In addition, TEM and STEM-HAADF measurements showed no direct correlations between the average Au particle size and catalytic properties. A number of nanoparticles in the range 1–7 nm with different distributions were observed in all catalysts (Table 6).

**Figure A1:**
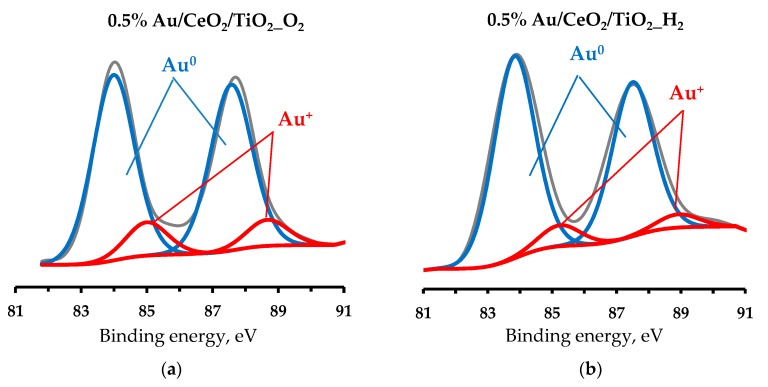

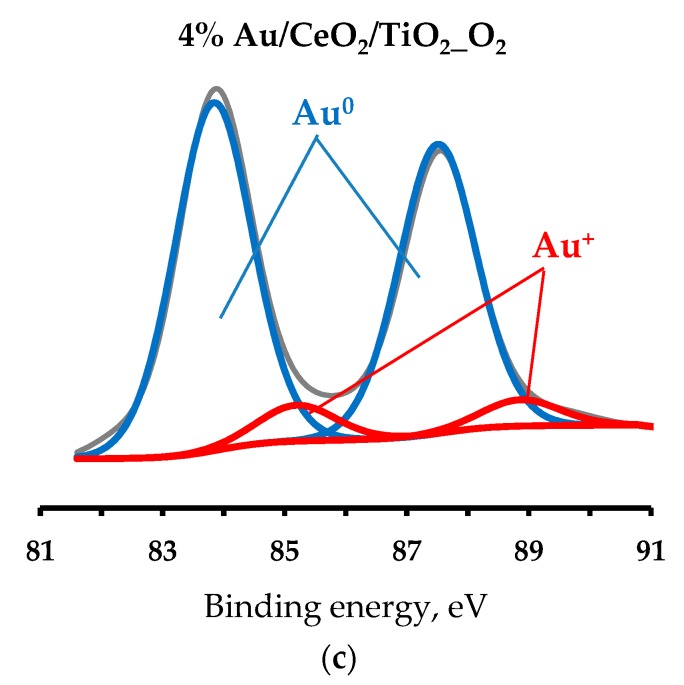
Au4f XP spectra of Au/CeO_2_/TiO_2_ samples with different gold content (0.5 or 4 wt.%) pretreated in H_2_ or O_2_ flow at 300 °C for 1 h: 0.5%Au/CeO_2_/TiO_2__O_2_ (**a**), 0.5%Au/CeO_2_/TiO_2__H_2_ (**b**) and 4%Au/CeO_2_/TiO_2__O_2_ (**c**).

XPS measurements of catalysts showed that gold formed different electronic states on the support surface—ions Au^+^ and Au^3+^, and neutral gold nanoparticles. The ratio among these states depends strongly on the support and the modifier nature (Table 7). XPS results for ceria modified samples are presented in Figure A1.

**Figure A2:**
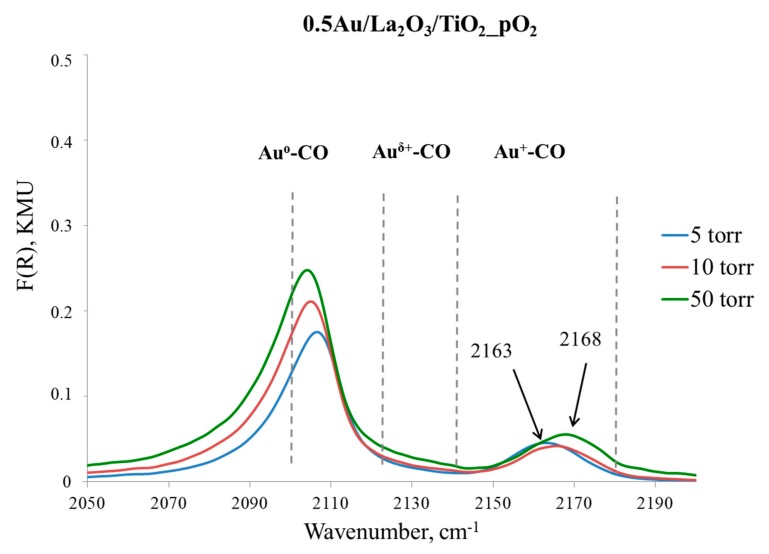

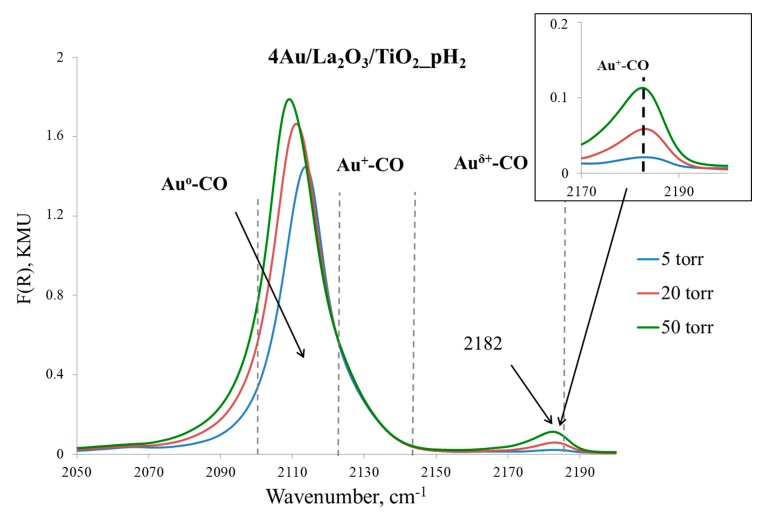
DRIFT spectra of CO adsorbed on the most active catalysts in peroxidative and aerobic oxidation of 1-phenylethanol. Reproduced from Ref. [43] with permission from the Royal Society of Chemistry, 2019.

According to the H_2_-TPR and CO_2_-TPD results, it was concluded that modification by La oxide favored the formation of very stable ionic species Au^δ+^ (0 < δ < 1) by their localization on the strong basic Lewis sites, formed by two-electron orbitals of the oxygen atom on the support surface (data not shown, obtained in [39]). The highest portion of such gold states was observed in the Au/La_2_O_3_/TiO_2_ sample, while they were practically absent in unmodified and modified with ceria catalysts. Also, according to DRIFT CO (Figure A2, Reproduced from Ref. [43] with permission from the Royal Society of Chemistry) and XPS data, the contribution of singly charged ions and their stability were maximum for the most active catalysts (4% Au/La_2_O_3_/TiO_2__H_2_ and 0.5% Au/La_2_O_3_/TiO_2__O_2_). Thus, although the amount of monovalent ions (determined by XPS) for some materials was comparable to the values found for lanthanum-modified catalysts, strong and stable gold ions were only found in lanthanum-modified samples, according to the applied DRIFT CO method [43].

**Figure 2:**
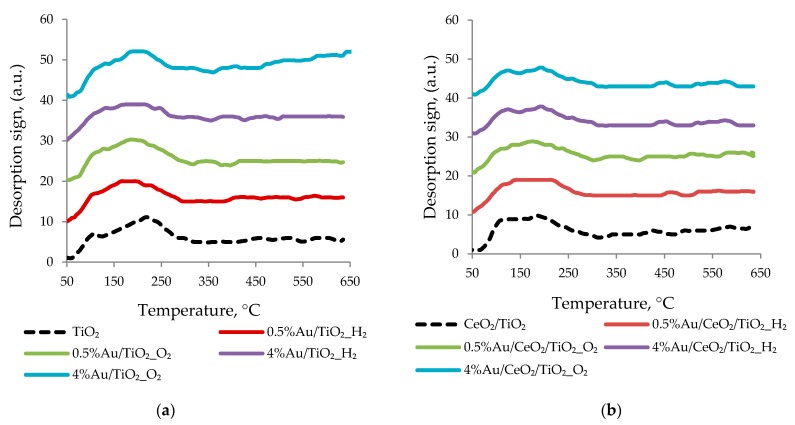

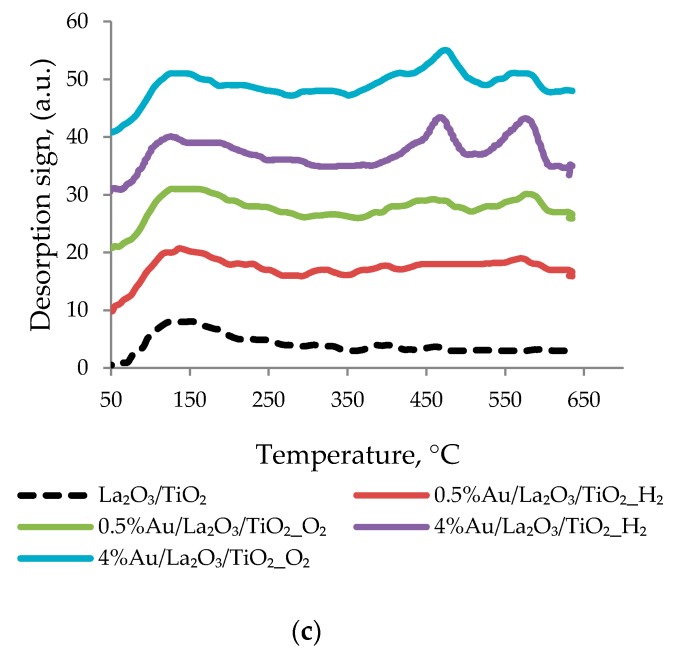
O_2_-TPD profiles for Au nanoparticles on TiO_2_ (**a**)CeO_2_/TiO_2_ (**b**)and La_2_O_3_/TiO_2_ (**c**)with different pretreatments.

O_2_-TPD was used to assess the nature of the interaction of oxygen with the surface of the catalyst and the support. The O_2_-TPD profiles obtained for the catalysts and their corresponding supports (Figure 2) show the presence of several peaks of oxygen desorption corresponding to different forms of adsorbed oxygen. 

Moreover, all the catalysts showed a wide peak of oxygen desorption in the range of 50–350 °C, which may be due to the adsorption of O_2_^−^on TiO_2_, according to [50]. It should be noted that pure titania showed three overlapping peaks (at 95, 205 and 292 °C) in this temperature range. After gold deposition on TiO_2_, a slight change in the shape of these peaks was observed. After titania was modified with ceria and lanthana, the shape and position of the wide peak in the low-temperature region changed, overlapping maxima were observed at 100, 190 and 265 °C for CeO_2_/TiO_2_ and at 110, 230 and 310 °C for La_2_O_3_/TiO_2_.

The deposition of gold on the ceria-modified support did not cause a significant change in the O_2_-TPD profiles. However, after the deposition of gold on the surface of lanthana-modified titania, high-temperature peaks appeared in the range of 400–600 °C, and catalysts with 4% Au had a noticeably high intensity, with peaks at 460 and 560 °C. These desorption peaks are likely to occur at higher temperatures and correspond to adsorbed O^−^ on the surface of TiO_2_, as described in [50,51]. Addition of lanthana and gold effectively stimulate the dissociation of O_2_ to O^−^, which has a higher activity than the super-oxo form O_2_^−^ in the oxidation reaction [50,52,53]. Thus, these results support the previously established favorable features of doping titania with lanthana (formation and stabilization of single charged gold ions through their localization on strong basic sites of La_2_O_3_/TiO_2_). Besides they revealed another promoting role of modifying additives of lanthana: providing the most active type of oxygen for effective oxidation of alcohols.

When comparing the XPS and the catalytic results of the catalysts in the peroxidative and aerobic oxidation of 1-phenylethanol (Table 8), there is no apparent correlation. Nevertheless, the most active catalysts (4% Au/La_2_O_3_/TiO_2__H_2_ and 0.5% Au/La_2_O_3_/TiO_2__O_2_) show the largest contribution of monovalent gold ions (Entries 1,2, Table 8).

### 3.3. Catalyst Recycling Tests

**Figure 3:**
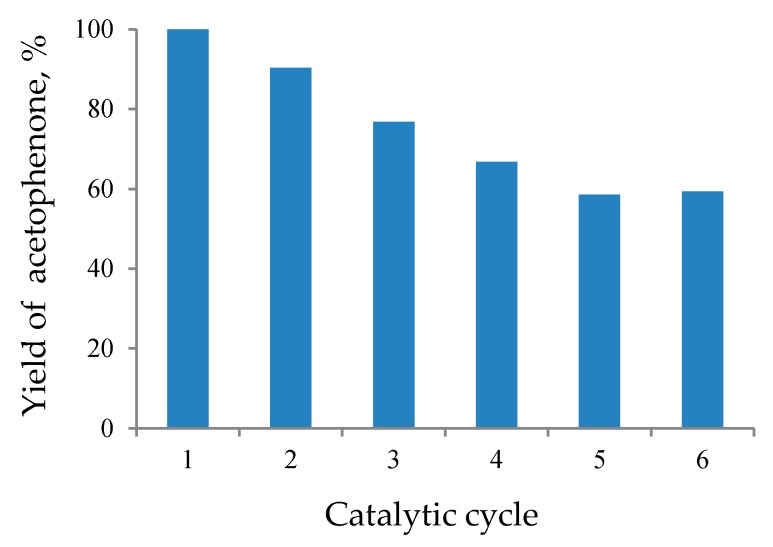
Effect of the catalyst recycling on the yield of acetophenone from peroxidative oxidation of 1-phenylethanol under catalyzed by 4% Au/La_2_O_3_/TiO_2__H_2_ (1.27 µmol Au, TBHP: 1-phenylethanol = 2.43,2 h, *T* = 80 °C).

The catalyst recyclability in the peroxidative oxidation of 1-phenylethanolwas investigated up to six consecutive cycles, as described in the Experimental part, with the best performing catalyst, i.e., 4% Au/La_2_O_3_/TiO_2__H_2_. As can be seen in Figure 3, there was a gradual catalyst deactivation during the recycling tests. Particularly, in the second cycle, the catalyst maintained 90% of activity, whereas in the third cycle a loss of 23% of its initial activity was observed. Consecutive decreasing of activity stopped at the sixth cycle where the yield of acetophenone kept the same level as in the fifth cycle (58–59%). Nevertheless, high selectivity to acetophenone was preserved in each cycle. 

**Figure 4:**
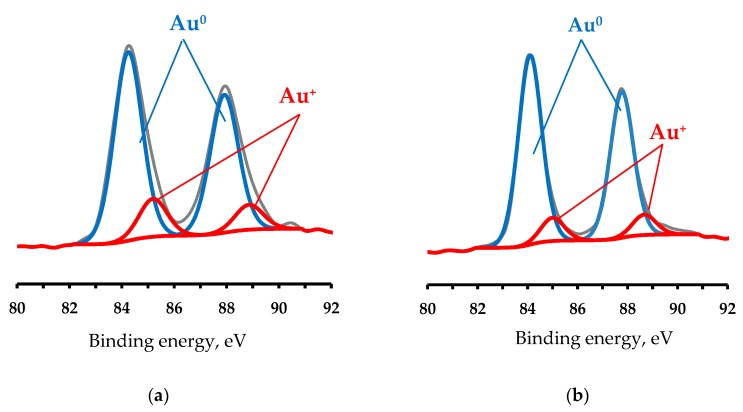
XPS of used 4%Au/La_2_O_3_/TiO_2__H_2_ catalyst after the 1st (**a**) and 6th (**b**) cycles of 1-phenylethanol peroxidative oxidation (reaction conditions as in Figure 1).

To find out the cause of the observed catalyst deactivation, XPS analysis of the catalyst after the first and last (6th) cycle was performed (Figure 4) and compared with the XPS results for the catalyst before reaction. As in the “fresh” catalyst, before reaction (Entry 1, Table 8), two electronic states of gold were found in the used catalysts: metallic (Au^0^) with binding energy (BE) (Au4f_7/2_) 84.2 and single charged ions (Au^+^) with BE (Au4f_7/2_) 85.2 eV in catalyst after the 1st cycle and Au^0^ with BE (Au4f_7/2_) 84.1 eV and (Au^+^) with BE (Au4f_7/2_) 85 eV [54,55,56,57] in catalyst after 6th cycles were detected. However, according to XPS measurements, the surface concentration of gold is different among the studied samples (Entries 1,3,4, Table 8). It can be seen from Table 8 that, with each cycle, loss of catalytic activity decreased proportionally to the contribution of monovalent gold. That can probably be the reason for deactivation, as also in the case of 1-octanol (see detailed information in Table A1 as well as discussion of previous results from [39]), since gold monovalent ions were the proposed active species for the reaction.

### 3.4. Quantum Chemical Simulation of the Alcohol Adsorption on a Gold Cluster

The purpose of this study was to understand the following issues relating to the nature of the active sites of the gold nanoparticles in the activation of phenylethanol at the atomic level:

(*i*)  Which phenylethanol coordination on a gold cluster is preferred (by OH– or C_6_H_5_– groups)?
(*ii*)  How do the structural features of the catalyst surface, including availability of low coordinated gold atoms, affect the adsorption of the alcohol?
(*iii*)  What is the effect of gold cationic sites on alcohol activation?

**Figure 5:**
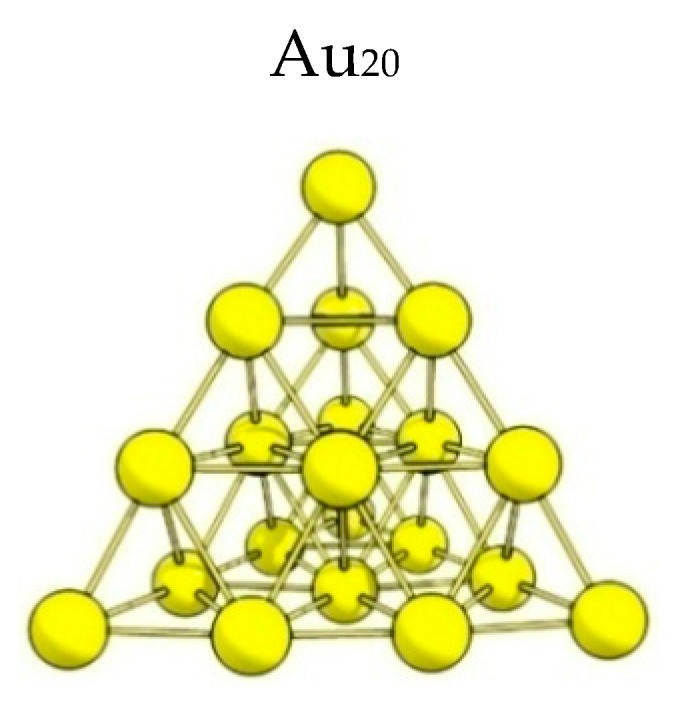

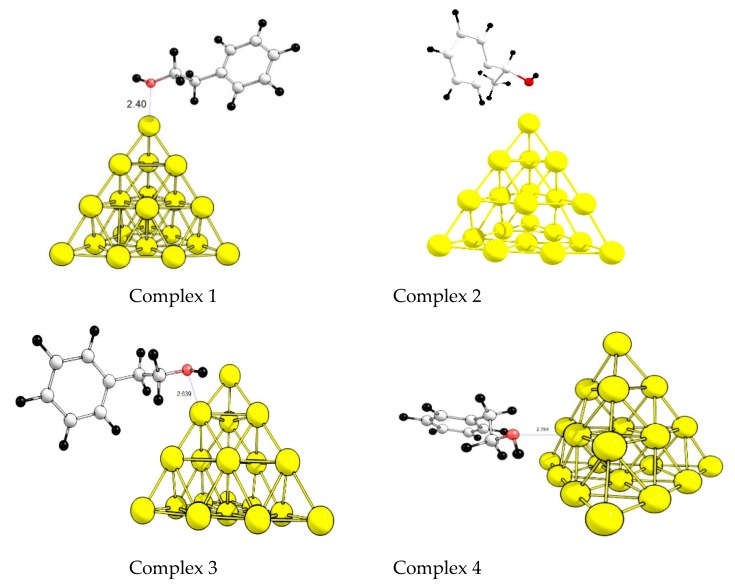
Optimized structures of Au_20_ clusters and phenylethanol-Au_20_ complexes (coordination by OH group).

The optimized structures of phenylethanol-Au_20_ complexes, in which the alcohol is coordinated to a gold atom by the OH group, are shown in Figure 5. The energy changes during the formation of the complexes and the corresponding standard enthalpies are collected in Table 9. According to the calculated data, the most favorable coordination during the interaction of phenylethanol with Au_20_ is carried out at the top of the cluster. Both 1-phenylethanol and 2-phenylethanol bind to the top gold atom with similar values of adsorption energy (57–60 kJ/mol). The binding energies of alcohol on the edge and facet gold atoms are significantly lower (considering 1-phenylethanol as an example).

**Figure 6:**
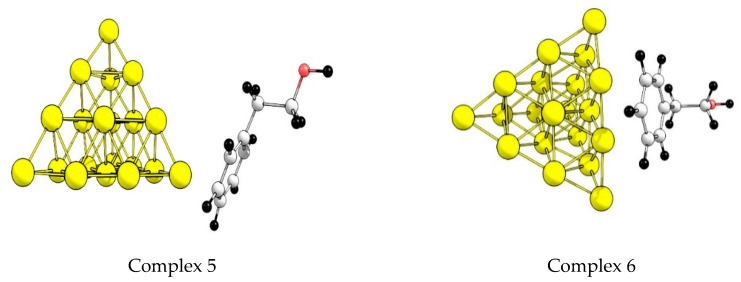
Optimized structures of phenylethanol-Au_20_ complexes (coordination by C_6_H_5_ fragment).

When alcohol is coordinated on the cluster through a benzene fragment (Figure 6), the adsorption energies decrease at all sites of Au_20_, compared to OH– group coordination. Among two different ways of alcohol coordination (OH– or C_6_H_5_–), coordination with the OH– group is advantageous (Complex 2, Figure 5). In this case, low-coordinated gold atoms are the most active in the activation of alcohol.

Then we examined how the gold cationic sites affect alcohol adsorption. The interaction of 1-phenylethanol or 2-phenylethanol with an Au_20_^+^ cluster was simulated at different coordinations (by the OH group or the phenyl fragment):

Au_20_^+^ + CH_3_–CH(OH)–Ph → (CH_3_–CH(OH)–Ph)Au_20_^+^ (4)

Au_20_^+^ + OH–CH_2_–CH_2_–Ph → (OH–CH_2_–CH_2_–Ph)Au_20_^+^ (5)

The optimized structures of phenylethanol-Au_20_^+^complexes have similar features with neutral phenylethanol-Au_20_. In contrast, the calculated energy changes in reaction 4 (Table 9) are larger than in reaction 1. The binding energy of phenylethanol to low-coordinated cationic gold atoms through the OH– group is significantly higher than on the neutral cluster. When 1-phenylethanol is coordinated by an aromatic fragment on Au_20_^+^, the adsorption energy increases and becomes almost the same as the coordination of alcohol with the OH group. Based on the study, it can be concluded that the cationic sites play an important role in phenylethanol adsorption.

## 4. Conclusions

From the above results, it could be concluded that Au/(Me*_x_*O*_y_*)/TiO_2_ systems were highly effective in the oxidation of 1-phenylethanol, and the catalysts modified with lanthana were the most active, as in the oxidation of 1-octanol. Furthermore, comparing our results on 1-phenylethanol oxidation with other gold supported catalysts (Table 1), it could be concluded that the Au/(Me*_x_*O*_y_*)/TiO_2_ systems are one of the most effective in peroxidative selective oxidation of 1-phenylethanol with TBHP, given the high alcohol/gold ratio (*R* = 5000), and low temperature (*T* = 80 °C) used without bases and solvents. 

In the absence of a solvent and using TBHP as an oxidizing agent, the most active catalysts are those with a high load of gold and the hydrogen treatment was beneficial. In the case of using solvent and oxygen as an oxidizing agent, as in the oxidation of 1-octanol, the catalysts with the lowest gold content after oxidative pretreatment showed the highest activity. The reason of such a difference most likely is the reduction of some part of unstable gold ions to metallic state in a 0.5% Au/La_2_O_3_/TiO_2__O_2_ catalyst by electron transfer under the influence of TBHP (even catalysts in «as prepared state» could provide high activity in 1-phenylethanol oxidation with TBHP after 6 h). Moreover, it was previously shown that there are the same contribution of stable gold ions in these two catalysts (0.5% Au/La_2_O_3_/TiO_2__O_2_ and 4% Au/La_2_O_3_/TiO_2__H_2_); furthermore, the difference in catalytic activity of 1-phenylethanol oxidation on these catalysts was not so noticeable. 

The most active catalysts had a high concentration of stable monovalent gold ions, and deactivation of catalysts in 1-phenylethanol oxidation is parallel to the reduction of Au^+^ (Au^δ+^) states, that confirms the cationic nature of the active sites. The promoting role of lanthanum additives consists not only in the formation of the most stable Au^+^ (Au^δ+^) species on the surface of La-modified TiO_2_ due to their localization at the strong basic Lewis sites, as proved earlier, but also in the presence of the most active type of oxygen, contributing to a more efficient oxidation of alcohols.

Thus, a general conclusion can be drawn that, despite the different nature of the studied alcohols, the nature of the active sites of Au/(Me*_x_*O*_y_*)/TiO_2_ catalysts in both the aerobic and peroxidative oxidation of 1-phenylethanol and 1-octanol is the same and monovalent gold ions are the active sites in these processes. Herein, the concentration, strength and stability of these sites are determined by the gold content, the nature of the support and modifier, and the pretreatment atmosphere.

The adsorption of 1-phenylethanol on Au clusters was simulated using DFT calculations. Among two different ways of alcohol coordination, that with the OH– group is the most advantageous one. In this case, low-coordinated gold atoms are the most active in the activation of alcohol. The binding energy of 1-phenylethanol with low-coordinated cationic gold atoms through the OH– group is significantly higher than that on the neutral cluster. Therefore, based on the quantum chemical calculations, it was concluded that the cationic sites play an important role in 1-phenylethanol adsorption, what confirms our suggestions on the gold cationic nature based on experimental results. The theoretical results were similar for 2-phenylethanol.

## Figures and Tables

**Table 1:** Catalytic oxidation of 1-phenylethanol using homogeneous and heterogeneous catalysts.

Catalyst	Oxidant	*P*, Atm	Solvent	*T*, °C	Reaction Time, h	^c^ *R*	^d^*Y_ac_*, %	Ref.
[Cu_2_(R)(CH_3_O)(NO_3_)]_2_(CH_3_O)_2_	TBHP	1	*-*	80	^b^ 1^MW^	71	54	[5]
^g^ [Cu(κONN’HL)(NO_3_) (*N*,*N*-dimethylformamide(DMF)](NO_3_)∙H_2_	TBHP	1	*-*	80	2	1000	12	[6]
^g^ [Cu(κONN’HL)(NO_3_)(DMF)] (NO_3_)∙H_2_+K_2_CO_3_	TBHP	1	*-*	80	2	1000	62	[6]
^h^ [Cu(1κNOO’,2κO’,3κO”L)]_n_	TBHP	1	*-*	120	^b^ 1^MW^	250	66	[7]
^h^ [Cu(1κNOO’,2κO’,3κO”L)]_n_ +2,2,6,6-Tetramethylpiperidin-1-yl)oxyl(TEMPO)	TBHP	1	*-*	120	^b^ 1^MW^	250	81	[7]
^h^ [Cu(κNOO’HL)Cl(CH_3_OH)]	TBHP	1	*-*	120	^b^ 1^MW^	250	82	[7]
^h^ [Cu(κNOO’HL)Cl(CH_3_OH)] +TEMPO	TBHP	1	*-*	120	^b^ 1^MW^	250	92	[7]
Carbon nanotubes (CNTs)	TBHP	1	*-*	80	^b^ 1^MW^	50	8	[8]
Graphene oxide (GO)	TBHP	1	*-*	80	^b^ 1^MW^	50	14	[8]
CoCl_2_	TBHP	1	*-*	80	^b^ 1^MW^	50	28	[8]
CoCl_2_–5%CNTs	TBHP	1	*-*	80	^b^ 1^MW^	50	85	[8]
CoCl_2_–5% GO	TBHP	1	*-*	80	^b^ 1^MW^	50	72	[8]
CuO	TBHP	1	*-*	80	^b^ 1^MW^	50	16	[8]
CuO–1%CNTs	TBHP	1	*-*	80	^b^ 1^MW^	50	59	[8]
Fe_2_O_3_	TBHP	1	*-*	80	^b^ 1^MW^	50	10	[8]
Fe_2_O_3_–1%CNTs	TBHP	1	*-*	80	^b^ 1^MW^	50	32	[8]
Fe_2_O_3_–CoCl_2_–5%CNTs	TBHP	1	*-*	80	^b^ 1^MW^	50	73	[8]
V_2_O_5_	TBHP	1	*-*	80	^b^ 1^MW^	50	45	[8]
CoCl_2_–V_2_O_5_–5%CNTs	TBHP	1	*-*	80	^b^ 1^MW^	50	54	[8]
[FeCl_2_(L)(2,20bipy)]	TBHP	1	*-*	150	^b^ 1^MW^	333	99	[9]
[FeCl_2_(L)(2,20bipy)]	TBHP	1	*-*	150	46	333	99	[9]
[Fe(bipy)_3_](CF_3_SO_3_)_2_	H_2_O_2_	1	CH_3_CN	100	0.5	100	62	[10]
[Fe(bipy)_3_](CF_3_SO_3_)_2_)+2-pyridinecarboxylic acid	H_2_O_2_	1	CH_3_CN	100	0.5	100	93	[10]
VOPO_4_+TEMPO	O_2_	4	H_2_O	80	6	20	^a^ 38.5(89)	[11]
NiO/SiO_2_	O_2_	1	*p*-xylen	100	6	12	51	[12]
MnO_2_ commercial	TBHP	1	^e^ ACN:tol	RT	7	1	84	[13]
MnO_2_ commercial	TBHP	1	^e^ ACN:tol	40	7	20	34	[13]
MnO_2_ commercial	H_2_O_2_	1	^e^ ACN:tol	40	5	20	0	[13]
MnO_2_ commercial	TBHP	1	^e^ ACN:tol	80	7	10	67	[13]
MnO_2_ commercial	-	1	^e^ ACN:tol	80	24	1	30	[13]
NbP–C	H_2_O_2_	1	CH_3_CN	90	24	11	^a^ 72	[14]
CeCrO_3_	TBHP	1	DMSO	90	6	10	^a^ 100	[15]
15wt.% Ag- Octahedral molecular sieve-2	TBHP	1	CH_3_CN	75	4	625	^a^ 71.5	[16]
0.9wt.% Pd/Aerosil380	O_2_	10	H_2_O	100	6	262	44.9	[17]
0.9wt.% Pd/Aerosil380	O_2_	10	H_2_O	100	12	262	75.1	[17]
1.0wt.% Pd/60wt.% Polyketone (PK)–SiO_2_	O_2_	10	H_2_O	100	6	262	62.2	[17]
1.0wt.% Pd/60wt.% PK–SiO_2_	O_2_	10	H_2_O	100	12	262	100	[17]
1.0wt.% Pd/76wt.% PK–SiO_2_	O_2_	10	H_2_O	100	6	262	58.3	[17]
1.0wt.% Pd/76wt.% PK–SiO_2_	O_2_	10	H_2_O	100	12	262	94.8	[17]
3wt.%Pd/O-Diamonds(Dia)	O_2_	1	*o*-xylen	100	4	1428	^a^ 27.9	[18]
3wt.%Pd/CeO_2_/O-Dia	O_2_	1	*o*-xylen	100	4	1428	^a^ 72.5	[18]
1.57wt.% Pd/CeO_2_	O_2_	1	-	120	2	649	^a^ 91	[19]
1.44wt.% Pd/apatite	O_2_	1	-	120	2	649	^a^ 90	[19]
10.10wt.% Ru/Mg–LaO	O_2_	1	toluene	80	4	10	96	[20]
10.10wt.% Ru/SiO_2_	O_2_	1	toluene	80	4	10	45	[20]
10.10wt.% Ru/Al_2_O_3_	O_2_	1	toluene	80	4	10	40	[20]
10.10wt.% Ru/MgO	O_2_	1	toluene	80	4	10	36	[20]
10.10wt.% Ru/TiO_2_	O_2_	1	toluene	80	4	10	36	[20]
1.54wt.% Au/CeO_2_	O_2_	1	-	120	2	649	95	[19]
1wt.% gold nanoparticles (Au NPs)/Ionic liquid(IL)/*N*-hydroxyphthalimide(NHPI)	O_2_	4	-	100	24	1356	^a^ 60(47)	[21]
1wt.% Au NPs/IL/NHPI	O_2_	4	-	160	24	6780	^a^ 77 (58)	[21]
1wt.%Au NPs– supported ionic liquid-like phases	H_2_O_2_	1	H_2_O	150	0.25	8	>90	[22]
1wt.% Au/Active carbon	TBHP	1	-	150	^b^ 2 ^MW^	500	55	[23]
1wt.% Au/carbon xerogel	TBHP	1	-	150	^b^ 2 ^MW^	500	90	[23]
1wt.% Au/Graphite	TBHP	1	-	150	^b^ 2 ^MW^	500	63	[23]
1wt.% Au/Microdiamonds	TBHP	1	-	150	^b^ 2 ^MW^	500	100	[23]
1wt.% Au/Nanodiamonds for liquid dispersion	TBHP	1	-	150	^b^ 2 ^MW^	500	83	[23]
1wt.%Au/Silicone carbide	TBHP	1	-	150	^b^ 2 ^MW^	500	73	[23]
0.89wt.% Au/Hydrotalcite (Ht)	Air	1	toluene	80	0.33	222	99	[24]
0.89wt.% Au/Ht	Air	1	toluene	40	3	222	99	[24]
0.89wt.% Au/Ht	Air	1	toluene	27	6	222	99	[24]
0.89wt.% Au/Al_2_O_3_	Air	1	toluene	27	3	222	71	[24]
0.89wt.% Au/MgO	Air	1	toluene	27	3	222	71	[24]
0.89wt.% Au/TiO_2_	Air	1	toluene	27	3	222	14	[24]
0.89wt.% Au/TiO_2_+Na_2_CO_3_	Air	1	toluene	27	3	222	65	[24]
0.89wt.% Au/SiO_2_	Air	1	toluene	27	3	222	<1	[24]
1.8wt.% Au/Layered double hydroxide	O_2_	1	toluene	80	2	200	99	[25]
1.0wt.% Au/Cu*_a_*Mg*_b_*Al*_c_*O*_x_*	O_2_	1	^f^ mes.	90	1	1181	85.1	[26]
5wt.% Au/TiO_2_	O_2_	1	-	120	6	500	99	[27]
5wt.% Au/Carbon black	O_2_	1	-	120	4	500	65	[27]
5wt.% Au/Single wall carbon nanotubes	O_2_	1	-	120	3	500	99	[27]
5wt.% Au/MnO_2_-R	O_2_	4	-	120	8	40,000	81	[28]
Au–Pd (2 wt.%, 1:1)/Sodium titanate nanotubes	Air	1	-	120	10	10,000	^a^ 84(86)	[29]
7.8wt.% Au/TiO_2_	O_2_	10	H_2_O	100	8	100	100	[30]
7.8wt.% Au/TiO_2_+ K_2_CO_3_	O_2_	10	H_2_O	100	2	100	93	[30]
10.83wt.% Au-dendrimers/ Mesoporous SiO_2_SBA-15+ 3 eq. K_3_PO_4_	O_2_	1	CH_2_Cl_2_/H_2_O	RT	24	33	99.1	[31]
0.5 wt.%(Au^0^–Pd^0^)/high surface area-BaAl_2_O_4_	O_2_	20	-	140	0.83	50,000	^a^ 97	[32]
4wt.% Au/La_2_O_3_/TiO_2_	TBHP	1	-	80	1	5000	98	This
4wt.% Au/La_2_O_3_/TiO_2_	O_2_	1	^f^ mes.	80	1	100	99	work

^a^ Instead of yield, data on conversion of alcohol are given, and acetophenone selectivity in brackets; ^b^ MW—microwave irradiation; ^c^ R—Alcohol/Active metal ratio (mol/mol); ^d^ Y_ac_—yield of acetophenone; ^e^ ACN:tol—CH_3_CN:toluene (3:1); ^f^ mes.—mesitylene; ^g^ [Cu(κONN’HL)(NO_3_) (DMF)](NO_3_)∙H_2_—copper complex with Schiff base ligand (HL) formed from salicylic aldehyde and aminoethylpiperazine (details on structure in [6]); ^h^ [Cu(1κNOO’,2κO’,3κO”-L)] (1) and [Cu(κNOO’-HL)Cl(CH_3_OH)] (2)—two aroylhydrazone Cu(II) complexes in two different tautomeric forms ((1) *enol* and (2) *keto*), H_2_L = 2-hydroxy(2-hydroxybenzylidene)benzohydrazide (details in [7]).

**Table 2:** Catalytic results of peroxidative oxidation of 1-phenylethanol ^1^.

Entry	Sample	Yield of Acetophenone (mol%) at Time (h)						
		0.25	0.5	1	2	3	4	6
1	-	0	0	1	2	3	3	4
2	TiO_2_	2	2	2	2	2	4	6
3	CeO_2_/TiO_2_	3	3	3	4	6	8	14
4	La_2_O_3_/TiO_2_	3	3	3	6	8	10	17
5	0.5% Au/TiO_2__H_2_	25	40	55	80	100	100	100
6	0.5% Au/TiO_2__O₂	18	30	43	69	81	98	100
7	0.5% Au/TiO_2__as	1	1	3	9	16	28	51
8	4% Au/TiO_2__H₂	33	46	75	100	100	100	100
9	4% Au/TiO_2__O₂	30	49	65	99	100	100	100
10	4% Au/TiO_2__as	1	2	6	19	28	45	82
11	0.5% Au/CeO_2_/TiO_2__H₂	27	37	60	85	100	100	100
12	0.5% Au/CeO_2_/TiO_2__O₂	28	36	72	90	100	100	100
13	0.5% Au/CeO_2_/TiO_2__as	2	3	3	7	14	28	61
14	4% Au/CeO_2_/TiO_2__H₂	36	49	85	100	100	100	100
15	4% Au/CeO_2_/TiO_2__O₂	22	41	75	100	100	100	100
16	4% Au/CeO_2_/TiO_2__as	3	4	10	25	46	67	100
17	0.5% Au/La_2_O_3_/TiO_2__H₂	31	45	55	88	100	100	100
18	0.5% Au/La_2_O_3_/TiO_2__O₂	38	49	61	96	100	100	100
19	0.5% Au/La_2_O_3_/TiO_2__as	1	1	2	8	18	42	84
20	4% Au/La_2_O_3_/TiO_2__H₂	41	55	98	100	100	100	100
21	4% Au/La_2_O_3_/TiO_2__O₂	22	40	67	100	100	100	100
22	4% Au/La_2_O_3_/TiO_2__as	1	2	6	29	54	100	100

^1^ Reaction conditions: TBHP: 1-phenylethanol = 2.43; *T* = 80 °C, stirring, *R*= 5000 (1.27 µmol of Au).

**Table 3:** Effect of the total gold amount on the peroxidative oxidation of 1-phenylethanol ^1^ using 4% Au/La_2_O_3_/TiO_2__O_2_.

Au Amount (µmol)	Yield of Acetophenone (mol%) at Time (min)					
	5	15	30	60	120	180
1.27	15	22	40	67	100	100
5	33	40	50	81	100	100
10	80	100	100	100	100	100
20	100	100	100	100	100	100

^1^ 1-phenylethanol (6 mmol), TBHP (70% aqueous solution, 14.6 mmol), *T* = 80 °C.

**Table 4:** Effect of alcohol/Au ratio (*R*) on aerobic oxidation of 1-phenylethanol ^1^ with 4% Au/La_2_O_3_/TiO_2__H_2_ catalyst.

Catalyst	*R*	Yield of Acetophenone, %
4% Au/La_2_O_3_/TiO_2__H₂	5000	0 ^2^
	500	50 ^2^
	100	98 ^3^

^1^ Reaction conditions: 0.1 M 1-phenylethanol in mesitylene, T = 80 °C, 30 mL/min O_2_. ^2^ After 6 h. ^3^ After 0.5 h.

**Table 5:** Catalytic results of aerobic oxidation of 1-phenylethanol ^1^.

Entry	Catalyst	Yield of Acetophenone (mol%) at Time (h)						
		0.25	0.5	1	2	3	4	6
1	0.5% Au/TiO_2__H₂	21	25	31	40	45	47	50
2	0.5% Au/TiO_2__O₂	26	32	38	44	49	52	58
3	4% Au/TiO_2__H₂	32	36	35	41	42	43	44
4	4% Au/TiO_2__O₂	19	21	25	30	35	38	40
5	0.5% Au/CeO_2_/TiO_2__H₂	45	50	60	65	68	70	72
6	0.5% Au/CeO_2_/TiO_2__O₂	70	86	96	99	100	100	100
7	4% Au/CeO_2_/TiO_2__H₂	53	59	65	69	71	73	78
8	4% Au/CeO_2_/TiO_2__O₂	40	44	50	55	59	65	70
9	0.5% Au/La_2_O_3_/TiO_2__H₂	90	95	97	100	100	100	100
10	0.5% Au/La_2_O_3_/TiO_2__O₂	95	98	100	100	100	100	100
11	4% Au/La_2_O_3_/TiO_2__H₂	88	96	99	100	100	100	100
12	4% Au/La_2_O_3_/TiO_2__O₂	79	88	93	97	98	99	100

^1^ Reaction conditions: *R* = 100, 0.1 M 1-phenylethanol in mesitylene; *T* = 80 °C, 30 mL/min O_2_, stirring = 800 rpm.

**Table 6:** Textural properties of supports and catalysts, analytical content and particle size of Au. Adapted from [38,39].

Sample	*S*_BET_, m^2^/g	EDX Au Content, wt.%	Au Average Particle Size, nm
TiO_2_	55	-	-
La_2_O_3_/TiO_2_	48	-	-
CeO_2_/TiO_2_	48	-	-
0.5% Au/TiO_2__H_2_	54	0.4	4.4
0.5% Au/TiO_2__O_2_	54	0.4	4.2
4% Au/TiO_2__H_2_	50	4.0	2.9
4% Au/TiO_2__O_2_	50	4.0	3.3
0.5% Au/CeO_2_/TiO_2__H_2_	47	0.3	3.4
0.5% Au/CeO_2_/TiO_2__O_2_	47	0.3	3.8
4% Au/CeO_2_/TiO_2__H_2_	46	4.1	2.8
4% Au/CeO_2_/TiO_2__O_2_	46	4.1	2.4
0.5% Au/La_2_O_3_/TiO_2__H_2_	47	0.5	2.8
0.5% Au/La_2_O_3_/TiO_2__O_2_	47	0.5	2.4
4% Au/La_2_O_3_/TiO_2__H_2_	43	3.3	2.6
4% Au/La_2_O_3_/TiO_2__O_2_	43	3.3	2.7

**Table 7:** Effect of gold content (0.5 or 4 wt.%) and redox treatment (H_2_ or O_2_) on contribution of different electron states of Au calculated according to XPS for Au/TiO_2,_ Au/CeO_2_/TiO_2_, Au/La_2_O_3_/TiO_2_ catalysts.

Catalyst	Au^(0, 1+ or 3+)^ Relative Content, %		
	Au^0^	Au^1+^	Au^3+^
^1^ 0.5% Au/TiO_2__H_2_	91	9	0
^1^ 0.5% Au/TiO_2__O_2_	84	16	0
^1^ 4% Au/TiO_2__H_2_	73	14	11
^1^ 4% Au/TiO_2__O_2_	89	11	0
^3^ 0.5% Au/CeO_2_/TiO_2__H_2_	91	9	0
^3^ 0.5% Au/CeO_2_/TiO_2__O_2_	85	15	0
^2^ 4% Au/CeO_2_/TiO_2__H_2_	68	20	12
^3^ 4% Au/CeO_2_/TiO_2__O_2_	90	10	0
^1^ 0.5% Au/La_2_O_3_/TiO_2__H_2_	80	20	0
^1^ 0.5% Au/La_2_O_3_/TiO_2__O_2_	65	35	0
^1^ 4% Au/La_2_O_3_/TiO_2__H_2_	81	19	0
^1^ 4% Au/La_2_O_3_/TiO_2__O_2_	83	17	0

^1^ Previous data from [38,39]. ^2^ Previous data from [42]. ^3^ XPS results firstly presented here.

**Table 8:** Catalytic results of the most active catalysts in the peroxidative and aerobic oxidation of 1-phenylethanol and contribution of various electronic states of gold in these catalysts, calculated by XPS.

Entry	Sample	Yield of Acetophenone in 2 h, mol %	Relative Au Content, %	
			Au^0^	Au^+^
1	4% Au/La_2_O_3_/TiO_2__H₂ ^1,a^	100	81	19
2	0.5% Au/La_2_O_3_/TiO_2__O₂ ^2,a^	100	65	35
3	4% Au/La_2_O_3_/TiO_2__H₂_1c ^1,b^	90	83	17
4	4% Au/La_2_O_3_/TiO_2__H₂_6c ^1,c^	59	89	11

^1^*R* = 5000, TBHP: 1-phenylethanol; *T* = 80 °C, stirring = 800 rpm. ^2^ *R* = 100, 0.1 M 1-phenylethanol in mesitylene; *T* = 80 °C, O_2_ = 30 mL/min, stirring = 800 rpm. ^a^ XPS performed for the catalyst before reaction. ^b^ XPS performed for the used catalyst after 1 cycle of reaction. ^c^ XPS performed for the used catalyst after 6 cycles of reaction.

**Table 9:** The calculated values of the energy change (Δ*E*, kJ/mol) and standard enthalpy (Δ*H*, kJ/mol) in the reactions of 1-phenylethanol (1) or of 2-phenylethanol (2) with the Au_20_^z^ cluster (z = 0, +1).

z	Complex	Isomer	Type of Coordination		Δ*E*	Δ*H*
0	1	2	OH–	top	−57	−54
0	2	1	OH–	top	−60	−57
0	3	2	OH–	edge	−33	−29
0	4	2	OH–	facet	−30	−31
0	5	2	C_6_H_5_–	top	−49	−45
0	6	2	C_6_H_5_–	edge	−23	−21
+1	1	2	OH–	top	−97	−92
+1	2	1	OH–	top	−112	−108
+1	3	2	OH–	edge	−75	−70
+1	4	2	OH–	facet	−56	−54
+1	5	2	C_6_H_5_–	top	−102	−100
+1	6	2	C_6_H_5_–	edge	−75	−72

## Appendix C

**Table A1:** Catalytic results of the most active catalyst in the aerobic oxidation of 1-octanol and contribution of various electronic states of gold in this catalyst, calculated by XPS. Adapted from [39].

Entry	Sample	Conversion of 1-Octanol After 6 h, ca.%	Relative Au Content, %	
			Au^0^	Au^+^
1	0.5% Au/La_2_O_3_/TiO_2__O₂ ^1^	63	65	35
2	0.5% Au/La_2_O_3_/TiO_2__O₂ ^2,a^	31	87	13
3	0.5% Au/La_2_O_3_/TiO_2__H₂ ^1,b^	34	87	13

^1^ XPS performed for the catalyst before reaction. ^2^ XPS performed for the catalyst after reaction. ^a^ XPS performed for the used catalyst after 6 h of reaction. ^b^ XPS performed for the catalyst pretreated in H_2_ at 500 °C for 1 h.

As we can see in Table A1, there was also significant a loss of 50% of its initial activity already in the second cycle of 1-octanol oxidation with O_2_ (Entry 2, Table A1). At the same time, a proportional decrease of monovalent ions in this sample after the first catalytic cycle is also observed. However, on the basis of H_2_-TPR analysis it was shown that such deactivation is related to the reduction of unstable monovalent gold ions (20–23%). Moreover, in order to check the stability of the mentioned gold ions and also confirm that they are the active sites, 0.5% Au/La_2_O_3_/TiO_2__O_2_ sample was treated in a hydrogen atmosphere at higher temperature (500 °C), for reduction of unstable gold ions. According to the XPS analysis of this sample (Entry 3, Table A1), it was found a decrease of 13% in the surface concentration of Au^δ+^ ions. This reduced sample was tested in a 1-octanol oxidation reaction. The initial and final conversion of 1-octanol on this sample was reduced almost two times, as the activity of the sample after the recycling test, where it was also observed 13% of gold monovalent ions.
